# The Prevention of Fire

**Published:** 1913-02-01

**Authors:** 


### The Prevention of Fire.

*To the Editor of* The Hospital.

Sir,—The information from Dr. Dobbie, of Toronto, in yours of the 25th inst., does not explain why the outbreak of fire at the hospital was not checked immediately it was discovered. He tells us the hospital was provided with hydrants, hose, fire-pails, and chemical fire-extinguishers, and that when the fire was discovered it was confined to a heating chamber, so that we conclude there was negligence in not immediately attacking the fire by means of the extinguishing appliances ready at hand. In our own case we had a fire on June 7, discovered one and three-quarter hour after all the employees had left, and when only one man was about, and he immediately turned on the fire hydrants and tackled the flames with such good effect that instead of £30,000 loss there was only a £2,000 loss, which exemplified the value of being prepared; but it is no credit to the hospital authorities, instead of immediately attacking the fire they run away and leave it to extend. Concerning his opinion of fire escapes, he evidently does not know that at North Grove Asylum, Hendon, a very large number of persons escaped by means of chute fire escapes, and this is only one of many instances where private fire escapes have been of great value.—Yours faithfully,

J. H. Heathman and Co.

London, January 21.

